# Prognostic Value of Preoperative Systemic Immune-Inflammation Index in Breast Cancer: A Propensity Score-Matching Study

**DOI:** 10.3389/fonc.2020.00580

**Published:** 2020-04-21

**Authors:** Xin Hua, Zhi-Qing Long, Yu-Ling Zhang, Wen Wen, Ling Guo, Wen Xia, Wen-Wen Zhang, Huan-Xin Lin

**Affiliations:** ^1^State Key Laboratory of Oncology in South China, Collaborative Innovation Center for Cancer Medicine, Sun Yat-sen University Cancer Center, Guangzhou, China; ^2^Jiangxi Provincial People's Hospital, Nanchang, China

**Keywords:** systemic immune-inflammation index, prognostication, breast cancer, survival, propensity score matching

## Abstract

**Purpose:** It was reported that the novel preoperative systemic immune-inflammation index (SII) can predict survival in cases of many malignant tumors. However, the prognostic significance of preoperative SII in breast cancer remains unclear. The purpose of this study was to investigate the relationship between SII and survival in breast cancer patients.

**Methods:** Breast cancer patients (1,026) who underwent a mastectomy at Sun Yat-sen University Cancer Center were retrospectively studied. The SII was determined using the following formula: neutrophil count × platelet count/lymphocyte count. The receiver operating characteristic (ROC) curve was used to determine the optimal cut-off value for SII. Propensity score matching (PSM) was applied to develop comparable cohorts of high SII group and low SII group.

**Results:** A total of 1,026 patients were included as the primary cohort, and 894 patients were matched and regarded as the matched cohort. Patients were divided into two groups based on SII value: SII <601.7 and high SII >601.7. In the primary cohort, the 5-years overall survival (OS), recurrence-free survival (RFS), and distant metastasis-free survival (DMFS) rates for high SII group and low SII group were (85.6% vs. 91.3%, *P* = 0.016), (95.8% vs. 96.4%, *P* = 0.684), and (83.5% vs. 90.6%, *P* = 0.007), respectively. Univariate analysis showed that histological type, T stage, N stage, PR, HER2, Ki67, and SII all showed significant associations with OS; and histological type, T stage, N stage, and SII all showed significant associations with DMFS. Multivariate survival analysis revealed that SII can independently predict OS (*P* = 0.017) and DMFS (*P* = 0.007). Similar results were found in PSM cohort.

**Conclusions:** Preoperative SII may be a reliable predictor of OS and DMFS in patients with operable breast cancer to provide personalized prognostication and assist in formulation of the clinical treatment strategy.

## Introduction

Breast cancer is the most common malignancy of women in China and worldwide (1, 2). The variable prognosis of breast cancer, as a heterogeneous tumor, is influenced by different genomic subtypes. Although substantial effort has been dedicated and tremendous advances have been made toward early detection and improvements in systemic therapy, the 5-year relative survival rate remains low (3). Therefore, patient stratification and individualized precision therapy strategy are needed.

Currently, well-recognized tumor-related histopathologic classification factors including, tumor size, stage, histological type and grade, lymph node status, hormone receptor (HR) status, and human epidermal growth factor receptor 2 (HER2) status are used to predict survival (4). However, they are usually available for assessment until postoperatively. To determine more reliable and easily obtained prognostic markers, numerous preoperative markers have been comprehensively evaluated in several studies (5, 6). Thus, far, there are few well-recognized preoperative biomarkers that have an independent prognostic value.

The cancer inflammation and the essential immune system role in cancer surveillance and elimination are valuable hallmarks of cancer (7, 8). Systemic inflammatory responses are involved at the molecular level in cancer formation and progression including DNA damage, tumor invasion, angiogenesis promotion, and migration (9–12). Furthermore, studies have confirmed that circulating lymphocytes are a potential indicator of the patient's inflammatory status (13). Studies examining the prognostic effects of inflammatory biomarkers in breast cancer have resulted in great improvements in recent years. C-reactive protein, lymphocyte-to-monocyte ratio (LMR), platelet-to-lymphocyte ratio (PLR), neutrophil-to-lymphocyte ratio (NLR), and the prognostic nutritional index have been confirmed as potential independent prognostic factors in breast cancer (5, 6, 14, 15). However, the prognostic value of the systemic immune-inflammation index (SII), combining lymphocyte, platelet, and neutrophil counts, has never been verified in breast cancer. Therefore, this study was aimed at determining the prognostic value of SII in breast cancer.

## Patients and Methods

### Patients

We retrospectively identified patients who underwent surgery for breast cancer from December 2010 to January 2012 at Sun Yat-sen University Cancer Center (SYSUCC), Guangzhou, China. Histopathological and clinical examinations data were obtained for all patients. The exclusion criteria were as follows: (1) male breast carcinoma; (2) ductal carcinoma *in situ* or distant metastasis; (3) treatment with neoadjuvant chemotherapy or radiotherapy; (4) acute and/or chronic inflammatory, hematologic, or autoimmune diseases; (5) use of immunosuppressive or anti-inflammatory medicines; and (6) loss of complete laboratory data. Tumor staging was based on the 7th edition of the AJCC TNM staging system for breast cancer. The expression of estrogen receptor (ER), progesterone receptor (PR), human epidermal growth factor receptor 2 (HER2), and Ki67 was scored based on the CAP/ASCO guidelines (16, 17). Systematic treatment and radiotherapy were based on National Comprehensive Cancer Network guidelines. Most of them received systematic therapy as adjuvant chemotherapy, endocrine treatment, or Trastuzumab for human epidermal growth factor receptor-2 (Her-2)-positive tumor. Radiotherapy of the chest wall and regional nodes was applied with a dose prescription of 50 Gy in 25 fractions. Patients receiving breast conserving surgery underwent RT of the whole breast up to a median dose of 50 Gy (range, 48–50 Gy) with 1.8–2 Gy/fraction. The median dose of the tumor bed boost was 10 Gy (range, 10–16 Gy).

### Data Collection and Definition

The primary preoperative laboratory data from within 3 days of the time of surgery and clinicopathological data were collected from the patients' medical records. SII was determined using the following formula: SII = P × N/L, where P, N, and L represent the platelet (10^9^/L), neutrophil (10^9^/L) and lymphocyte (10^9^/L) counts, respectively.

### Follow-Up

The patients were followed up carefully by conducting an outpatient examination or a telephonic interview. Overall survival (OS), recurrence-free survival (RFS), distant metastasis-free survival (DMFS) were defined as the time from the date of diagnosis to the date of death/first event (recurrence, distant metastasis) or last follow-up.

### Statistical Analysis

Statistical analyses were performed using the SPSS 23.0 (SPSS, Inc., Chicago, IL) and GraphPad Prism 6.0 software (GraphPad, La Jolla, CA). The best cut-off value of SII was calculated by receiver operating characteristic (ROC) curve analysis use the highest Youden's index for predicting survival status. Propensity score matching of 1:3 scheme with a caliper width equal to 0.2 was applied to develop comparable cohorts of patients with low SII value and high SII value. Covariates for matching included age, histological type, T stage, N stage, clinical stage, ER, PR, HER2, and KI67. Association between categorical variables was analyzed using the Chi-square or Fisher exact test. Continuous variables were compared using Mann-Whitney test. The relation between NLR, PLR, and SII was examined with linear regression. Survival curves were calculated using the Kaplan-Meier method and compared via the log-rank test. Simple and multivariate regression analyses were performed using the Cox proportional hazards model and multivariate regression analyses model for variables with *P* < 0.10 in the univariate analysis. Two-sided *P* < 0.05 were considered statistically significant.

## Results

### Patient Characteristics

**Figure 1:**
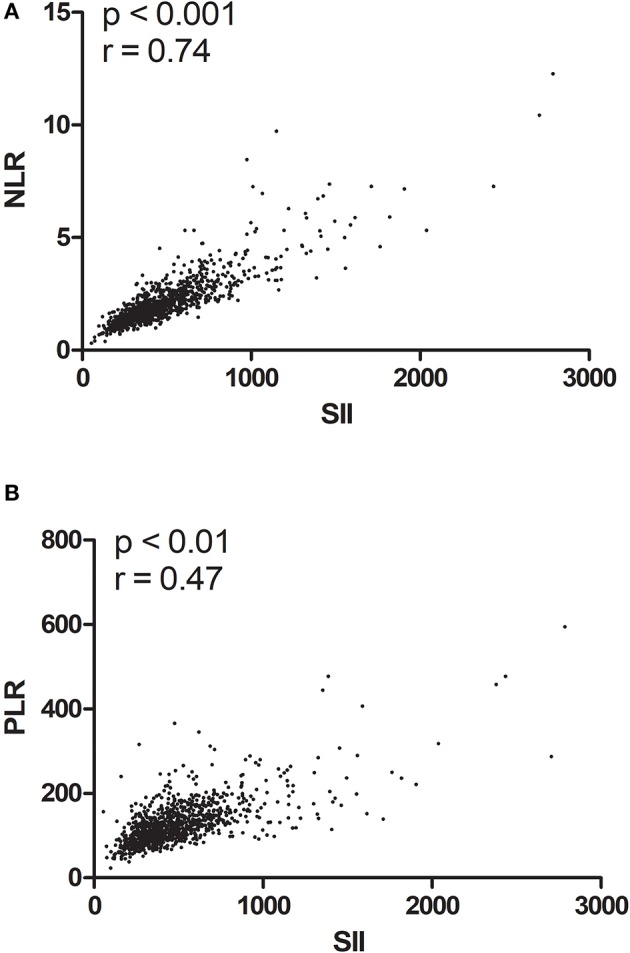
Linear regression association between SII and NLR, PLR. Linear regression association curves for: **(A)** SII and NLR; **(B)** SII and PLR.

**Figure 2:**
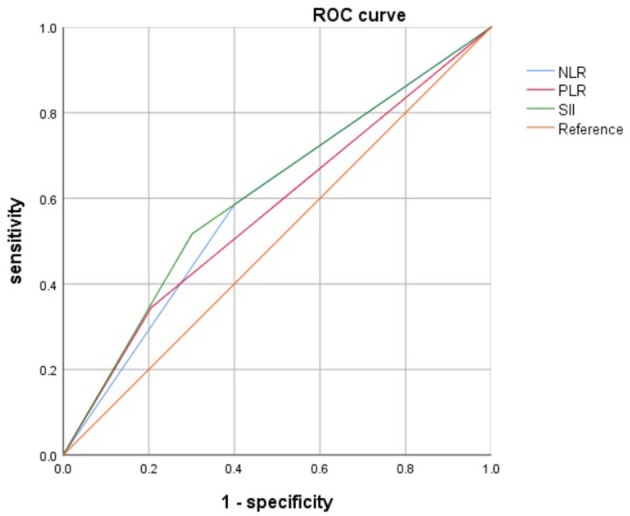
Receiver operating characteristic (ROC) curve analysis of SII and NLR, PLR.

The baseline characteristics of the 1,026 patients included in this study are shown in Table 1. Clinical stage I, II, and III disease was noted in 238 (23.2%), 541 (52.7%), and 247 (24.1%) patients, respectively. The median patient age was 47 years (range 22–87 years), and the median follow-up period was 68.5 months (range 0.9–87.5 months). The median OS was 65.8 months, while the 5-year OS rate was 91.5%. During the final follow-up, 101 (9.8%) patients died and 925 (90.2%) were still alive. Patients were stratified by the best cut-off SII value of 601.7 (low, <601.7; high, >601.7) and 782 patients showed low SII values whereas 244 patients had high SII values. We analyzed the association between SII and pre-surgical inflammations of NLR and PLR, and there is a linear association between SII and NLR (*P* < 0.001, *r* = 0.74; Figure 1A) and PLR (*P* < 0.01, *r* = 0.47; Figure 1B). Then, we further compared the prognostic value of these three and found that SII was better than NLR and PLR (SII AUC = 0.608, NLR AUC = 0.593, PLR = 0.569; Figure 2).

**Table 1:** Clinicopathologic characteristics before and after matching.

**Characteristic**	**Primary cohort**			**PSM cohort**		
	**Low SII**	**High SII**	***P*-value**	**Low SII**	**High SII**	***P*-value**
Total	782	244		656	238	
Age (years)			0.003			0.576
≥60	132 (12.9%)	22 (2.1%)		71 (7.9%)	22 (24.6%)	
<60	650 (63.4%)	222 (21.6%)		585 (65.4%)	216 (24.2%)	
Histological type			0.159			0.985
Invasive ductal carcinoma	693 (67.5%)	224 (21.8%)		603 (67.4%)	218 (24.4%)	
Others	89 (8.7%)	20 (1.9%)		53 (5.9%)	20 (2.2%)	
T stage^#^			0.052			0.897
1	263 (25.6%)	71 (6.9%)		205 (22.9%)	71 (7.9%)	
2	449 (43.8%)	141 (13.7%)		386 (43.2%)	141 (15.8%)	
3	38 (3.7%)	18 (1.8%)		36 (4.0%)	16 (1.8%)	
4	32 (3.1%)	14 (1.4%)		29 (3.2%)	10 (1.1%)	
N stage^#^			0.583			0.965
0	414 (40.4%)	121 (11.8%)		331 (37.0%)	118 (13.2%)	
1	195 (19.0%)	68 (6.6%)		168 (18.8%)	65 (7.3%)	
2	101 (9.8%)	32 (3.1%)		91 (10.2%)	32 (3.6%)	
3	72 (7.0%)	23 (2.2%)		66 (7.4%)	23 (2.6%)	
Clinical stage^#^			0.091			0.925
I	190 (18.5%)	48 (4.7%)		140 (15.7%)	48 (5.4%)	
II	411 (40.1%)	130 (12.7%)		351 (39.3%)	130 (14.5%)	
III	181 (17.6%)	66 (6.4%)		165 (18.5%)	60 (6.7%)	
ER			0.838			0.714
Negative	220 (21.4%)	67 (6.5%)		172 (19.2%)	66 (7.4%)	
Positive	562 (54.8%)	177 (17.3%)		484 (54.1%)	172 (19.2%)	
PR			0.319			0.794
Negative	287 (28.0%)	81 (7.9%)		223 (24.9%)	78 (8.7%)	
Positive	495 (48.2%)	163 (15.9%)		433 (48.4%)	160 (17.9%)	
HER2						0.555
Negative	536 (52.2%)	165 (16.1%)	0.788	460 (51.5%)	162 (18.1%)	
Positive	246 (24.0%)	79 (7.7%)		196 (21.9%)	76 (8.5%)	
Ki67			0.934			0.641
Negative	249 (24.3%)	77 (7.5%)		212 (23.7%)	73 (8.2%)	
Positive	533 (51.9%)	167 (16.3%)		444 (49.7%)	165 (18.5%)	
Molecular subtype			0.606			0.142
Luminal A	189 (18.4%)	55 (5.4%)		163 (18.2%)	54 (6.0%)	
Luminal B/HER2–	272 (26.5%)	88 (8.6%)		238 (26.6%)	87 (7.8%)	
Luminal B/HER2+	189 (18.4%)	54 (5.3%)		159 (17.8%)	51 (5.7%)	
HER2 enriched	57 (5.6%)	25 (2.4%)		37 (4.1%)	25 (2.8%)	
Triple negative	75 (7.3%)	22 (2.1%)		59 (6.6%)	21 (2.3%)	

ER, estrogen receptor; PR, progesterone receptor; HER2, human epidermal growth factor receptor-2; PSM, propensity score matching; SII, systemic immune-inflammation index.

# *According to the 7th edition of the UICC/AJCC staging system*.

The characteristics of two groups were similar, except for the age (*P* = 0.003) and T stage (*P* = 0.052). Therefore, we established a new cohort using propensity score matching (PSM) to avoid potentially confounding the findings. After PSM, 894 patients were identified, and the clinical characteristics among the two groups were well-balanced (all *P* > 0.05; Table 1).

### Prognosis Value of SII

**Figure 3:**
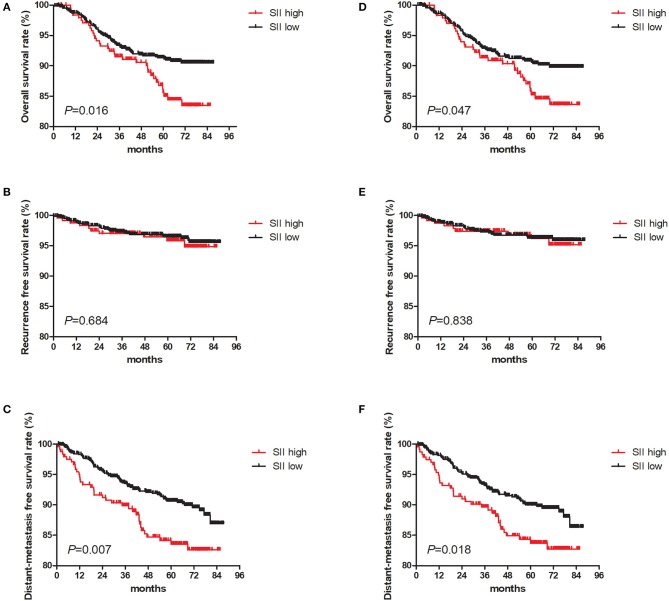
Kaplan Meier survival curves for overall survival (OS), recurrence-free survival (RFS) and distant metastasis-free survival (DMFS) in primary cohort and propensity score matching (PSM) cohort. Kaplan-Meier curves for: **(A)** overall survival in primary cohort; **(B)** overall survival in PSM cohort; **(C)** recurrence-free survival in primary cohort; **(D)** recurrence-free survival in PSM cohort; **(E)** distant metastasis-free survival in primary cohort; **(F)** distant metastasis-free survival in PSM cohort.

The 5-year OS rate for the entire cohort was 89.9%, and in the primary cohort, the corresponding values for patients in high SII group was significantly shorter than those in low SII group (85.6%, and 91.3%, respectively, *P* = 0.016, Figure 3A). The 5-year RFS rate for the entire cohort was 96.3%, and the corresponding values was comparable between two groups (95.8 and 96.4%, respectively; *P* = 0.684, Figure 3B). The 5-years DMFS rate for the entire cohort was 88.9%, and this corresponding values was significantly shorter in the high SII group (83.5%) than in the low SII group (90.6%; *P* = 0.007; Figure 3C). Then, we reanalyzed in the PSM cohort and revealed similar differences in the prognosis of the OS (*P* = 0.047) (Figure 3D), RFS (*P* = 0.838; Figure 3E), and DMFS (*P* = 0.018; Figure 3F).

### Simple and Multivariate Cox Regression Analysis for OS and DMFS

Simple Cox regression analysis in the primary cohort revealed that histological type, T stage, N stage, PR, HER2, Ki67, molecular subtype, and SII all showed significant associations with OS; and histological type, T stage, N stage, and SII all showed significant associations with DMFS. Multivariate survival analysis revealed that SII can independently predict OS (*P* = 0.019; Table 2) and DMFS (*P* = 0.008; Table 3). Similar results were demonstrated in the PSM cohort (Tables 2, 3).

**Table 2:** Univariate and multivariate analyses of overall survival.

**Characteristic**	**Primary cohort**				**PSM cohort**			
	**Univariate analysis**		**Multivariate Cox regression analysis**		**Univariate analysis**		**Multivariate Cox regression analysis**	
	**Hazard ratio (95% CI)**	***P***	**Hazard ratio (95% CI)**	***P***	**Hazard ratio (95% CI)**	***P***	**Hazard ratio (95% CI)**	***P***
Age (years)	1.140 (0.677–1.920)	0.621			1.414 (0.787–2.541)	0.247		
Histological type	6.011 (1.482–24.371)	0.012^*^	3.522 (0.861–14.409)	0.080	8.392 (1.170–60.213)	0.034^*^	5.294 (0.734–38.202)	0.098
T stage^#^	2.068 (1.668–2.564)	<0.001^*^	1.204 (0.936–1.550)	0.148	2.107 (1.676–2.649)	<0.001^*^	1.197 (0.916–1.563)	0.187
N stage^#^	2.508 (2.108–2.984)	<0.001^*^	2.373 (1.947–2.892)	<0.001^*^	2.528 (2.103–3.039)	<0.001^*^	2.398 (1.947–2.954)	<0.001^*^
ER	0.665 (0.443–1.000)	0.050	0.791 (0.447–1.399)	0.421	0.658 (0.429–1.009)	0.055	0.789 (0.434–1.435)	0.438
PR	0.493 (0.333–0.728)	<0.001^*^	0.642 (0.397–1.040)	0.072	0.462 (0.307–0.693)	<0.001^*^	0.599 (0.365–0.984)	0.043^*^
HER2	1.622 (1.092–2.409)	0.017^*^	0.883 (0.566–1.376)	0.582	1.594 (1.053–2.415)	0.028^*^	0.842 (0.526–1.347)	0.473
Ki67	2.403 (1.427–4.047)	0.001^*^	1.688 (0.964–2.958)	0.067	2.351 (1.372–4.028)	0.002^*^	1.720 (0.962–3.076)	0.067
Molecular subtype	1.337 (1.152–1.553)	<0.001^*^	1.106 (0.834–1.468)	0.484	1.341 (1.147–1.68)	<0.001^*^	1.065 (0.793–1.430)	0.677
SII	1.651 (1.093–2.495)	0.017^*^	1.646 (1.085–2.496)	0.019^*^	1.534 (1.003–2.346)	0.048^*^	1.611 (1.049–2.474)	0.030^*^

A Cox proportional hazards model was used to conduct multivariate analyses. All variables were transformed into categorical variables. HRs were calculated for age (≥60 y vs. <60 y); histological type (invasive ductal carcinoma vs. others); T stage (T3-4 vs. T1-2); N stage (N2-3 vs. N0-1); ER (negative vs. positive); PR (negative vs. positive); HER2 (negative vs. positive); Ki67 (negative vs. positive); molecular subtype (others vs. luminal) and SII (high vs. low). We selected variables using the backward stepwise approach. The P-value threshold was 0.10 (P ≥ 0.10) for the removal of insignificant variables from the model.

* P < 0.05: ER, estrogen receptor; PR, progesterone receptor; HER2, human epidermal growth factor receptor-2; PSM, propensity score matching; SII, systemic immune-inflammation index.

# *According to the 7th edition of the UICC/AJCC staging system*.

**Table 3:** Univariate and multivariate analyses of distant metastasis-free survival.

**Characteristic**	**Primary cohort**				**PSM cohort**			
	**Univariate analysis**		**Multivariate Cox regression analysis**		**Univariate analysis**		**Multivariate Cox regression analysis**	
	**Hazard ratio (95% CI)**	***P***	**Hazard ratio (95% CI)**	***P***	**Hazard ratio (95% CI)**	***P***	**Hazard ratio (95% CI)**	***P***
Age (years)	0.697 (0.382–1.269)	0.237			0.779 (0.378–1.605)	0.498		
Histological type	3.337 (1.229–9.058)	0.018^*^	2.485 (0.913–6.765)	0.075	4.658 (1.148–18.892)	0.031^*^	3.429 (0.841–13.969)	0.086
T stage^#^	1.788 (1.436–2.227)	<0.001^*^	1.197 (0.935–1.532)	0.154	1.817 (1.435–2.300)	<0.001^*^	1.242 (0.953–1.620)	0.109
N stage^#^	2.014 (1.713–2.368)	<0.001^*^	1.875 (1.565–2.246)	<0.001^*^	1.945 (1.639–2.308)	<0.001^*^	1.788 (1.477–2.165)	<0.001^*^
ER	0.990 (0.650–1.507)	0.962			0.990 (0.632–1.549)	0.964		
PR	0.824 (0.560–1.212)	0.324			0.780 (0.519–1.173)	0.233		
HER2	1.412 (0.958–2.080)	0.081	1.120 (0.756–1.659)	0.571	1.482 (0.986–2.228)	0.058	1.098 (0.723–1.666)	0.661
Ki67	1.431 (0.931–2.199)	0.102			1.554 (0.981–2.461)	0.060	1.329 (0.835–2.115)	0.230
Molecular subtype	1.002 (0.856–1.174)	0.976			1.031 (0.873–1.217)	0.722		
SII	1.721 (1.157–2.559)	0.007^*^	1.720 (1.156–2.561)	0.008^*^	1.635 (1.085–2.463)	0.019^*^	1.727 (1.144–2.607)	0.009^*^

A Cox proportional hazards model was used to conduct multivariate analyses. All variables were transformed into categorical variables. HRs were calculated for age (≥60 y vs. <60 y); histological type (invasive ductal carcinoma vs. others); T stage (T3-4 vs. T1-2); N stage (N2-3 vs. N0-1); ER (negative vs. positive); PR (negative vs. positive); HER2 (negative vs. positive); Ki67 (negative vs. positive); molecular subtype (others vs. luminal) and SII (high vs. low). We selected variables using the backward stepwise approach. The P-value threshold was 0.10 (P ≥ 0.10) for the removal of insignificant variables from the model.

* P < 0.05: ER, estrogen receptor; PR, progesterone receptor; HER2, human epidermal growth factor receptor-2; PSM, propensity score matching; SII, systemic immune-inflammation index.

# *According to the 7th edition of the UICC/AJCC staging system*.

## Discussion

In recent years, many studies have investigated SII in various cancers. Thus, far, there is no study explored the prognostic significance of SII in breast cancer. To our knowledge, this is the first study to date of patients with operable breast cancer, and our results demonstrated that the SII value was an independent marker of survival and may better than NLR and PLR in breast cancer patients.

Recent studies have shown that the systemic inflammatory response can induce malignant tumor behavior and lead to shorter survival durations in patients with malignant solid tumors, and can also predict the prognosis of cancer (18–21). SII as a novel systemic immune-inflammation index, that represents the overall inflammatory, and immune status of the patient has proven to be a meaningful preoperative biomarker in various tumors (18, 20–22). Hu et al. found that in hepatocellular carcinoma SII was associated with circulating tumor cell levels and local recurrence (21), while Wang et al. noted that SII was associated with age, tumor invasion, local lymph node metastasis, and distant metastasis in gastric cancer (18). Similar results were also obtained for small cell lung cancer and cancer (22). However, the prognostic value of SII in breast cancer remains unclear.

Many studies have reported that preoperative inflammation indexes are effective biomarkers in predicting the prognosis in breast cancer (5, 6). Bárbara et al. found that the PLR and NLR were related to clinical outcomes in breast cancer patients (5). Hideya et al. revealed that the PLR, NLR, and LMR all significantly predict survival in breast cancer and that the prognostic efficiency of the PLR was better than those of the NLR and LMR (14). The present study also demonstrated that SII can independently predict survival in patients with breast cancer. As a more convenient, more accessible, lower cost, non-invasive prognostic indicator, SII can serve as a supplement to TNM staging and a prognostic predictor for breast cancer patients.

The mechanisms underlying the prognostic significance of SII for the OS in breast cancer remain unclear, and the physiopathologic role of platelets, neutrophils, and lymphocytes might explain this to some extent. By activating the NF-κB and TGF-β/Smad pathways synchronously, platelet-derived TGF-β and direct platelet-tumor cell contacts can induce mesenchymal-like transition and promote metastasis in cancer cells (23, 24). Neutrophils can help cancer cells escape immune surveillance by promoting cancer cell invasion, proliferation, and metastasis (25, 26). Lymphocytes affect tumor growth by secreting cytokines and inducing cytotoxic cell death while inhibiting the proliferation and migration of cancer cells (27). These existing mechanisms suggest that a higher SII essentially implies a stronger inflammatory response, but a weaker immune defense in cancer patients, resulting in a lower survival rate. SII, which comprehensively reflects the immune, and inflammatory status of the host (21), promises to be a useful predictor of cancer prognosis in clinical practice. Our research firmly supports this hypothesis. Moreover, further studies need to be conducted to identify the novel mechanisms in the future.

The findings of this study may help clinicians gain a better understanding of the relationship between immunization, inflammation, and cancer. These results may also provide clinicians with a guideline to develop appropriate therapeutic regimens for individualized precision therapy in breast cancer patients. Breast cancer patients showing high preoperative SII values should receive complementary immunotherapy and anti-inflammatory agents like the active anti-inflammatory agent in Thymus vulgaris, Herceptin, and aspirin, which should be administered immediately after surgery.

There are several limitations of this study. Firstly, as a retrospective study with a relatively small sample size obtained at a single center, the conclusions drawn from the current study may be biased and we are actively seeking cooperation from other centers to verify the results. Secondly, although SII is an independent predictor of breast cancer prognosis, its sensitivity, and specificity are not very high, indicating that further prospective studies are required to determine the appropriate cut-off value. Thirdly, the fact that we have collected some survival information over the phone may have introduced information bias. Finally, different surgical procedures may affect inflammatory indicators and inflammatory indicators may also change before and after surgery (28–30). In view of the limitation of existing data (only 185 patients underwent breast-conserving surgery), we will further study the influence of different surgical procedures and changes in preoperative and postoperative inflammatory indicators on the prognosis in subsequent studies.

## Conclusions

In summary, the current results confirm that SII is a prospective biomarker to predict the clinical outcome in breast cancer. High SII values indicate a higher risk of mortality and distant metastasis among breast cancer patients.

## Data Availability Statement

The datasets generated for this study are available in the RDD repository with the accession number RDDA2020001442 (http://www.researchdata.org.cn/).

## Ethics Statement

The studies involving human participants were reviewed and approved by the Clinical Research Ethics Committee of SYSUCC (approval number: GZR2017-224). The patients/participants provided their written informed consent to participate in this study.

## Author Contributions

XH: conceptualization. XH and Y-LZ: methodology. XH and Z-QL: software. WW and Z-QL: validation. XH, WX, and Y-LZ: formal analysis. WW and Y-LZ: investigation. H-XL, WX, and W-WZ: resources. XH and H-XL: data curation. XH: writing (original draft preparation). WX and LG: visualization. WX, W-WZ, and H-XL: supervision. H-XL: project administration. LG and H-XL: funding acquisition. All authors: writing (review and editing), approved the version to be published, and agreed to be accountable for all aspects of the work in ensuring that questions related to the accuracy or integrity of any part of the work are appropriately investigated and resolved.

## Conflict of Interest

The authors declare that the research was conducted in the absence of any commercial or financial relationships that could be construed as a potential conflict of interest.

## Abbreviations

SII: systemic immune-inflammation index
ROC: receiver operating characteristic
PSM: Propensity score matching
ER: estrogen receptor
PR: progesterone receptor
OS: overall survival
HR: hormone receptor
HER2: human epidermal growth factor receptor 2
LMR: lymphocyte-to-monocyte ratio
PLR: platelet-to-lymphocyte ratio
NLR: neutrophil-to-lymphocyte ratio.

## References

[1] Siegel RL Miller KD Jemal A Cancer statistics, 2015. CA Cancer J Clin. (2015) 65:5–29. 10.3322/caac.21254 25559415

[2] Chen W Zheng R Baade PD Zhang S Zeng H Bray F. Cancer statistics in China, 2015. CA Cancer J Clin. (2016) 66:115–32. 10.3322/caac.21338 26808342

[3] Goldhirsch A Winer EP Coates AS Gelber RD Piccart-Gebhart M Thürlimann B. Personalizing the treatment of women with early breast cancer: highlights of the St Gallen international expert consensus on the primary therapy of early breast cancer 2013. Ann Oncol. (2013) 24:2206–23. 10.1093/annonc/mdt303 23917950 PMC3755334

[4] Lal P Tan LK Chen B. Correlation of HER-2 status with estrogen and progesterone receptors and histologic features in 3,655 invasive breast carcinomas. Am J Clin Pathol. (2005) 123:541–6. 10.1309/YMJ3A83TB39MRUT9 15743737

[5] Wariss BR de Souza Abrahao K de Aguiar SS Bergmann A Santos Thuler LC. Effectiveness of four inflammatory markers in predicting prognosis in 2374 women with breast cancer. Maturitas. (2017) 101:51–6. 10.1016/j.maturitas.2017.04.015 28539169

[6] Cho U Park HS Im SY Yoo CY Jung JH Suh YJ. Prognostic value of systemic inflammatory markers and development of a nomogram in breast cancer. PLoS One. (2018) 13:e0200936. 10.1371/journal.pone.0200936 30048474 PMC6062056

[7] Hanahan D Weinberg RA. Hallmarks of cancer: the next generation. Cell. (2011) 144:646–74. 10.1016/j.cell.2011.02.013 21376230

[8] Grivennikov SI Greten FR Karin M. Immunity, inflammation, and cancer. Cell. (2010) 140:883–99. 10.1016/j.cell.2010.01.025 20303878 PMC2866629

[9] Hernandez M Martin R Garcia-Cubillas MD Maeso-Hernández P Luisa Nieto M. Secreted PLA2 induces proliferation in astrocytoma through the EGF receptor: another inflammation-cancer link. Neuro Oncol. (2010) 12:1014–23. 10.1093/neuonc/noq078 20639215 PMC3018927

[10] Dai J Lu Y Roca H Keller JM Zhang J McCauley LK. Immune mediators in the tumor microenvironment of prostate cancer. Chin J Cancer. (2017) 36:29. 10.1186/s40880-017-0198-3 28292326 PMC5351274

[11] Nguyen AV Wu YY Liu Q Wang D Nguyen S Loh R. STAT3 in epithelial cells regulates inflammation and tumor progression to malignant state in colon. Neoplasia. (2013) 15:998–1008. 10.1593/neo.13952 24027425 PMC3769879

[12] Nguyen AV Wu YY Lin EY. STAT3 and sphingosine-1-phosphate in inflammation-associated colorectal cancer. World J Gastroenterol. (2014) 20:10279–87. 10.3748/wjg.v20.i30.10279 25132744 PMC4130835

[13] Wang L Shen Y. Imbalance of circulating T-lymphocyte subpopulation in gastric cancer patients correlated with performance status. Clin Lab. (2013) 59:429–33. 10.7754/Clin.Lab.2012.120625 23724636

[14] Takeuchi H Kawanaka H Fukuyama S Kubo N Hiroshige S Yano T. Comparison of the prognostic values of preoperative inflammation-based parameters in patients with breast cancer. PLoS ONE. (2017) 12:e0177137. 10.1371/journal.pone.0177137 28489884 PMC5425200

[15] Mohri T Mohri Y Shigemori T Takeuchi K Itoh Y Kato T. Impact of prognostic nutritional index on long-term outcomes in patients with breast cancer. World J Surg Oncol. (2016) 14:170. 10.1186/s12957-016-0920-7 27349744 PMC4924248

[16] Reisenbichler ES Lester SC Richardson AL Dillon DA Ly A Brock JE. Interobserver concordance in implementing the (2010). ASCO/CAP recommendations for reporting ER in breast carcinomas: a demonstration of the difficulties of consistently reporting low levels of ER expression by manual quantification. Am J Clin Pathol. (2013) 140:487–94. 10.1309/AJCP1RF9FUIZRDPI 24045544

[17] Rakha EA Starczynski J Lee AH Ellis IO. The updated ASCO/CAP guideline recommendations for HER2 testing in the management of invasive breast cancer: a critical review of their implications for routine practice. Histopathology. (2014) 64:609–15. 10.1111/his.12357 24382093

[18] Wang K Diao F Ye Z Zhang X Zhai E Ren H. Prognostic value of systemic immune-inflammation index in patients with gastric cancer. Chin J Cancer. (2017) 36:75. 10.1186/s40880-017-0243-2 28899420 PMC5596912

[19] Aziz MH Sideras K Aziz NA Mauff K Haen R Roos D. The systemic-immune-inflammation index independently predicts survival and recurrence in resectable pancreatic cancer and its prognostic value depends on Bilirubin levels: a retrospective multicenter cohort study. Ann Surg. (2018) 270:139-46. 10.1097/SLA.0000000000002660 29334554

[20] Fankhauser CD Sander S Roth L Gross O Eberli D Sulser T. Systemic inflammatory markers have independent prognostic value in patients with metastatic testicular germ cell tumours undergoing first-line chemotherapy. Br J Cancer. (2018) 118:825–30. 10.1038/bjc.2017.467 29485982 PMC5877429

[21] Hu B Yang XR Xu Y Sun Y-F Sun C Guo W. Systemic immune-inflammation index predicts prognosis of patients after curative resection for hepatocellular carcinoma. Clin Cancer Res. (2014) 20:6212–22. 10.1158/1078-0432.CCR-14-0442 25271081

[22] Gao Y Zhang H Li Y Wang D Ma Y Chen Q. Preoperative increased systemic immune-inflammation index predicts poor prognosis in patients with operable non-small cell lung cancer. Clin Chim Acta. (2018) 484:272–7. 10.1016/j.cca.2018.05.059 29860033

[23] Labelle M Begum S Hynes RO. Direct signaling between platelets and cancer cells induces an epithelial-mesenchymal-like transition and promotes metastasis. Cancer Cell. (2011) 20:576–90. 10.1016/j.ccr.2011.09.009 22094253 PMC3487108

[24] Stanger BZ Kahn ML. Platelets and tumor cells: a new form of border control. Cancer Cell. (2013) 24:9–11. 10.1016/j.ccr.2013.06.009 23845439 PMC3743536

[25] Mantovani A Allavena P Sica A Balkwill F. Cancer-related inflammation. Nature. (2008) 454:436–44. 10.1038/nature07205 18650914

[26] Mantovani A Cassatella MA Costantini C Jaillon S. Neutrophils in the activation and regulation of innate and adaptive immunity. Nat Rev Immunol. (2011) 11:519–31. 10.1038/nri3024 21785456

[27] Ferrone C Dranoff G. Dual roles for immunity in gastrointestinal cancers. J Clin Oncol. (2010) 28:4045–51. 10.1200/JCO.2010.27.9992 20644090 PMC4872327

[28] Vanni G Materazzo M Perretta T Ambrogi V Claudio Mineo T Pompeo E. Impact of Awake breast cancer surgery on postoperative lymphocyte responses. In Vivo. (2019) 33:1879–84. 10.21873/invivo.11681 31662515 PMC6899130

[29] Mineo TC Sellitri F Vanni G Gallina FT Ambrogi V. Immunological and inflammatory impact of non-intubated lung metastasectomy. Int J Mol Sci. (2017) 18:1466. 10.3390/ijms18071466 28686211 PMC5535957

[30] Lin JX Wang ZK Huang YQ Xie J-W Wang J-B Lu J. Dynamic changes in pre- and postoperative levels of inflammatory markers and their effects on the prognosis of patients with gastric cancer. J Gastrointest Surg. (2020). 10.1007/s11605-020-04523-8. [Epub ahead of print]. PMC7904717 32016671

